# Nitric oxide implication in the control of seed dormancy and germination

**DOI:** 10.3389/fpls.2013.00346

**Published:** 2013-09-19

**Authors:** Erwann Arc, Marc Galland, Béatrice Godin, Gwendal Cueff, Loïc Rajjou

**Affiliations:** ^1^INRA, Institut Jean-Pierre Bourgin (UMR1318 Institut National de la Recherche Agronomique – AgroParisTech), Laboratory of Excellence “Saclay Plant Sciences”, VersaillesFrance; ^2^AgroParisTech, UFR de Physiologie végétaleParis, France; ^3^University of Innsbruck, Institute of BotanyInnsbruck, Austria

**Keywords:** abscisic acid, dormancy, germination, nitric oxide, seed, vigor

## Abstract

Germination ability is regulated by a combination of environmental and endogenous signals with both synergistic and antagonistic effects. Nitric oxide (NO) is a potent dormancy-releasing agent in many species, including *Arabidopsis*, and has been suggested to behave as an endogenous regulator of this physiological blockage. Distinct reports have also highlighted a positive impact of NO on seed germination under sub-optimal conditions. However, its molecular mode of action in the context of seed biology remains poorly documented. This review aims to focus on the implications of this radical in the control of seed dormancy and germination. The consequences of NO chemistry on the investigations on both its signaling and its targets in seeds are discussed. NO-dependent protein post-translational modifications are proposed as a key mechanism underlying NO signaling during early seed germination.

## INTRODUCTION

Survival of plant species mainly relies on the sexual reproduction which gives rise to new populations. During this process, the adult angiosperm plants produce flowers which upon fertilization give rise to seeds, the main unit of dispersal of flowering plants. In the plant life cycle, the seed and seedling stages are key developmental stages conditioning the final yield of crops. Indeed, seed dormancy, viability, and germination vigor are among the main concerns for agricultural productivity. High vigor seed lots display a low dormancy and lead to seedlings able to withstand extreme stress conditions. If not completely released, dormancy will negatively influence seed germination, which is detrimental to crop yield. However, from an agronomical point of view, lack of dormancy is not a desirable trait as it may lead to pre-harvest sprouting (Bewley and Black, 1994). Therefore, the management of this trait is of fundamental concern for the seed industry and agriculture performance. Thus, investigation of seed quality, toward a better understanding of dormancy, germination and longevity, is of paramount agronomical importance. All these seed features are complex traits controlled by a large number of genes, which are affected by both developmental and environmental factors.

Numerous distinct nitrogen-containing compounds have been shown to positively influence seed germination especially by releasing seed dormancy and improving seed vigor in a wide range of species (Bethke et al., 2007b). These concentration-dependent effects could allow the sensing of the presence of these essential resources in the direct environment. The possibility that all these molecules could act in a similar way prompted plant biologists to look for a possible common nitrogen-containing intermediate and pinpointed nitric oxide (NO) as a possible candidate. Indeed, since its discovery, this radical has progressively emerged as an ubiquitous molecule in both animal and plant signaling networks (Baudouin, 2011). Increasing reports highlight its large implication in diverse signaling pathways regulating growth and developmental processes all along the plant life cycle. A key role for NO was further demonstrated in plant response to abiotic and biotic stresses. Instead of describing in details all these roles that have already been extensively discussed in recent reviews (Besson-Bard et al., 2008; Wilson et al., 2008; Moreau et al., 2010; Baudouin, 2011), we will focus on the implications of NO in the control of seed dormancy and germination with a particular emphasis on the experiments carried out on the model Angiosperm plant *Arabidopsis* thaliana. The present review also aims to provide outlooks for future investigation in this field.

## DEFINITION AND GENERAL OVERVIEW ON SEED DORMANCY AND GERMINATION

### SEED DORMANCY

Under natural conditions, an appropriate timing of seed germination is determinant to ensure optimal growth conditions for the young seedlings and guarantee the survival of the species (Bewley, 1997). Seed dormancy is one of the mechanisms contributing to this spatio-temporal adjustment and is defined as a block to the completion of germination of an intact viable seed placed under (temporary) favorable conditions in an otherwise unfavorable season (Bewley, 1997; Finch-Savage and Leubner-Metzger, 2006; Graeber et al., 2012). It may be due to certain properties of the seed coat, mobilization of reserve components, hormone levels, or the joint action of several of these factors (Koornneef et al., 2002). Thus, dormancy is determined by genetic factors but it can also be substantially modulated by environmental parameters (Graeber et al., 2012). Indeed, the alleviation of this blockage can be conditioned by several distinct environmental (temperature, humidity, light, nutrient concentration⋯) or physical (testa rupture⋯) factors. The exact conditions required for dormancy release and subsequent germination depend on the species and thus contribute to the adequacy of the plant to its environment by delaying germination until the seed meets appropriate conditions for its development. In addition, the depth of primary dormancy in mature seeds can depend on the conditions under which the mother plant was exposed such as temperature or availability of mineral elements (such as nitrate) in the soil (Alboresi et al., 2005; Kendall et al., 2011). Thus, seeds have developed a complex control of the depth of dormancy integrating diverse spatio-temporal parameters allowing a dynamic definition of the minimal requirements for germination. In addition, when a non-dormant seed encounters inappropriate conditions for germination, it can enter into a so-called secondary dormancy. Overall, these mechanisms contribute to the sensing of environmental conditions and can lead to dormancy cycling under natural conditions (Footitt et al., 2011).

Abscisic acid (ABA) is considered as the pivotal hormone responsible for the induction and maintenance of seed dormancy (Nambara et al., 2010). ABA is accumulated during seed maturation reaching high levels in dry seeds. Dry dormant seeds were found to contain higher amounts of ABA than dry after-ripened non-dormant seeds (Ali-Rachedi et al., 2004). Upon imbibition, a significant decrease in ABA content was observed in both dormant and non-dormant seeds (Ali-Rachedi et al., 2004). However, after 3 days of imbibition a significant up-accumulation of ABA was detected in dormant seeds only. Exposition of dormant seeds to common dormancy-releasing treatments such as cold-stratification or exogenous nitrate supply leaded to ABA levels similar to non-dormant seeds and prevented the increase in ABA observed when dormancy is maintained (Ali-Rachedi et al., 2004). Reactive oxygen species (ROS) and NO counteract the positive effect of ABA on seed dormancy maintenance. Exogenous application of fluridone (an inhibitor of ABA synthesis) also efficiently released seed dormancy by reducing ABA levels highlighting the requirement for de novo ABA synthesis for the maintenance of this blockage and the existence of a dynamic equilibrium between ABA synthesis and catabolism during seed imbibition (Ali-Rachedi et al., 2004). In addition, recent experiments demonstrated that two independent dormancy-releasing treatments led to similar proteome adjustments supporting the occurrence of shared molecular mechanisms underpinning seed dormancy release (Arc et al., 2012). Furthermore, recent data emphasize the importance of redox control of seed proteome in dormancy release (Marx et al., 2003; Bykova et al., 2011a,b). Thus, ROS and NO appear as good candidates, acting synergistically to release dormancy, putatively acting upstream of ABA.

### SEED GERMINATION

Seed germination is temporally defined as the sequence of molecular and physiological events initiated upon imbibition of non-dormant seed and leading to the radicle protrusion through the seed external envelopes (testa and endosperm) that marks the end of germination *sensu stricto* (Bewley, 1997). Seed germination constitutes a pivotal physiological transition and is associated with a strong modification of the transcriptome (~one-third of the genome) and metabolism over a short time period (around 36–48 h for non-dormant *Arabidopsis* seeds) relatively to the plant life cycle. During this process, the initially quiescent dry seed successively go through three major steps of water uptake (Bewley, 1997; Weitbrecht et al., 2011). The first step consists in a rapid imbibition of the initially quiescent seeds that lead to the progressive resumption of metabolic activity, gene expression (transcription), protein synthesis and processing and DNA repair (Weitbrecht et al., 2011). The recapitulation of the metabolic activity mainly depends on the stored proteins and metabolites. The importance of the compounds accumulated in the seeds during the maturation was further highlighted by the finding that stored mRNAs and proteins are sufficient for germination *sensu stricto* (Rajjou et al., 2004; Sano et al., 2012). *De novo* protein synthesis from the stored mRNAs occurs during the very early step of germination. During this period, the proteins translated are similar to those accumulated during the late maturation and already abundant in seeds reflecting an early recapitulation of the corresponding gene expression program during early germination (Rajjou et al., 2006, 2012). During the second step of water uptake, the water content only slightly increases while important metabolic changes take place inside the seeds. A significant shift is observed during this step from maturation to germination program of development that includes the preparation for seedling establishment (Lopez-Molina et al., 2002; Nonogaki et al., 2007). This two steps time course is consistent with a model proposing that recapitulation of the late maturation program occurs during early germination up to an ABA-dependent developmental checkpoint after which the seed can either activate its germination program or maintain a dormant state notably depending on the sensing of environmental conditions during early imbibition (Lopez-Molina et al., 2002; Rajjou et al., 2012). During this period, seeds maintain their desiccation tolerance. At the end of this second step, if the “decision” to pursue toward germination is taken, the growth potential of the embryo progressively overcome the mechanical constraints imposed by the surrounding layers leading to the successive rupture of the testa and the endosperm (Nonogaki, 2006; Bentsink and Koornneef, 2008). The protrusion of the radicle through the seed coat is thus achieved as a result of important cell elongation without any cell division (Sliwinska et al., 2009) and occurs concomitantly with an important resumption of water uptake. The ABA/gibberellins (GAs) balance coordinate this last step with a decrease in ABA leading to the progressive release its inhibitory effect on endosperm rupture while an important increase in bioactive GAs levels both enhanced the growth potential of the embryo and induced hydrolytic enzymes that weaken the barrier tissues (Bewley and Black, 1994; Muller et al., 2006; Finkelstein et al., 2008).

### GERMINATION VIGOR

If the seed encounters suitable conditions for germination during its life, it may, if still viable, allow the young seedling establishment. But as a consequence of aging, the seed germination vigor can be severely affected. In other words, the capacity of a seed lot to germinate rapidly, uniformly and in a wide range of environmental conditions can be impaired or destroyed. As the seed germination process mainly relies on stored mRNA and proteins (Rajjou et al., 2004), damages at the DNA level can result in an aborted development of the seedling. Thus, cellular repair mechanisms especially at the DNA level but also for certain protein post-translational modifications (PTMs) play an essential role in seed vigor (Rajjou et al., 2012). Due to seed high vulnerability to injury, abiotic, and biotic stresses during imbibition, germination is considered as the most critical phase of the plant life cycle. The level of reactive oxygen and nitrogen species (respectively ROS and RNS), influenced by the storage and environmental conditions will determine a balance between the required signaling events and the detrimental oxidative damages (Bailly et al., 2008; Rajjou et al., 2008, 2012; Arc et al., 2011).

## NITROGEN OXIDES IMPLICATION IN THE CONTROL OF SEED DORMANCY AND GERMINATION

### NITRATE AND NITRITE AVAILABILITY: DETERMINANT FACTORS FOR SEED DORMANCY RELEASE AND SUBSEQUENT GERMINATION

Nitrate (${NO}_{3}^{-}$) is considered as a major nitrogen source for most plant species. Nitrate reduction into nitrite (${NO}_{2}^{-}$) is catalyzed by nitrate reductase (NR) that produces nitrogen-containing metabolites, such as amino acids and NO. Apart from being an essential nutrient, nitrate is also considered as a signaling molecule involved in both plant metabolism regulation and developmental processes (Krouk et al., 2010). In particular, nitrate has been shown to promote seed dormancy release and subsequent germination in numerous plant species (Bewley and Black, 1994). Most of the first experiments mainly investigated the effect of nitrate on these physiological processes although the principal product of its assimilation, nitrite can also alleviate seed dormancy (Bethke et al., 2006a).

Exogenous treatments with nitrates were shown to promote seed germination in *Arabidopsis* by reducing the light requirement (Hilhorst and Karssen, 1988; Batak et al., 2002). The enhancement of germination mediated by light absorbed by phytochrome-A operates via the very-low-fluence response (VLFR; Botto et al., 1996). Thus, nitrate could stimulate the accumulation of cGMP, which then promotes some phytochrome responses (Ludidi and Gehring, 2003). Moreover, a positive correlation between endogenous or applied nitrate levels and germination response to ethylene or GAs was reported for *Chenopodium album* seeds (Saini et al., 1985). In *Arabidopsis*, high nitrate feeding of mother plants is associated with higher nitrate content and lower dormancy of the seed progeny (Alboresi et al., 2005). This result suggests a negative correlation between nitrate levels in dry mature seeds and the depth of dormancy. In addition, mutation in the nitrate transporter NRT1.1/CHL1 resulted in lower sensitivity to exogenous nitrate indicating that this protein may be required for nitrate uptake by the seed (Alboresi et al., 2005). Moreover, mutants in the seed specific nitrate transporter AtNRT2.7, involved in nitrate loading into the vacuole during seed maturation, displayed reduced nitrate content and slightly increased dormancy (Chopin et al., 2007). Overall, nitrate availability in seeds appears as an important determinant of seed dormancy.

The reduced dormancy of NR deficient seeds, impaired in nitrate assimilation, along with the finding that glutamine, another nitrogen source did not affect seed germination suggest that the effect of nitrate is unrelated to plant nutrition (Alboresi et al., 2005). As stated in the previous part, exogenous nitrate application was proved to negatively affect ABA content during *Arabidopsis* seed imbibition (Ali-Rachedi et al., 2004). In addition, controlled nitrate supply to the mother plants led to ABA contents negatively correlated to the endogenous nitrate concentration in dry mature seeds (Matakiadis et al., 2009). Accordingly, it has recently been demonstrated that the gene expression of the ABA catabolic enzyme, CYP707A2, was positively regulated by both endogenous and exogenous nitrate (Matakiadis et al., 2009). Thus, the positive effect of nitrate on dormancy alleviation is presumably mediated by affecting ABA metabolism.

### NITRIC OXIDE, THE KEY SIGNALING ELEMENT MEDIATING NITRATE RESPONSE IN SEEDS?

Nitric oxide is a gaseous diatomic free radical detected at low levels in the atmosphere. It is also present in the soils at a concentration depending on the micro-biotic environment (Simontacchi et al., 2007). Moreover, nitrogen fertilization was shown to increase NO release from the soils and proposed to account for the fitness of nitrogen-fertilized plants (Lamattina et al., 2003). NO was shown to efficiently break the dormancy and / or promote germination of several orthodox seeds (Beligni and Lamattina, 2000; Bethke et al., 2004b; Sarath et al., 2006; Liu et al., 2007; Gniazdowska et al., 2010a) including in *Arabidopsis thaliana* (Bethke et al., 2006b).

#### Nitric oxide: a key mediator of seed dormancy release

Recent data disclosed that the improvement of dormant-seeds germination provided by exogenous treatments with various nitrogenous molecules, including nitrate, and nitrite, most presumably occurs through NO production (Bethke et al., 2004b, 2006a). Accordingly, the NO content in homogenates from 24 h-imbibed soybean and sorghum embryonic axes, detected by electron paramagnetic resonance (EPR)-spin trapping, increased with increasing nitrate supply during seed imbibition (Caro and Puntarulo, 1999; Simontacchi et al., 2004). This result pinpoints exogenous nitrate concentration during seed imbibition as a key determinant of NO release.

Indeed, NO is well known to release seed dormancy in numerous species (Bethke et al., 2007b). For instance, pharmacological approaches demonstrated that most known NO donors promoted dormancy alleviation and subsequent germination while NO scavengers favored dormancy maintenance and counteracted the positive effect of NO donors (Bethke et al., 2007a). In addition, it has been shown that NO may alleviate dormancy of apple embryos *via* a transient accumulation of ROS, leading to enhanced ethylene emission as required to terminate germination *sensu stricto* (Gniazdowska et al., 2007, 2010a,b). NO also proved efficient to reverse blue light inhibition of dormant wheat seed germination, presumably acting interdependently with methyl-jasmonates in controlling reduction of ABA (Jacobsen et al., 2013).

In tomato seeds, the NO scavenger, carboxy-2-phenyl-4,4,5-tetramethylimidazole-1-oxyl 3-oxide (cPTIO), was shown to prevent germination stimulation by fluridone, an ABA synthesis inhibitor (Piterkova et al., 2012). On the contrary, exogenous sodium nitroprusside (SNP), commonly used as NO donor, enhanced the positive effect of norfluorazon, another ABA synthesis inhibitor, on dormancy release of *Arabidopsis* C24 seeds (Bethke et al., 2006b). Moreover, SNP was shown to reduce seed sensitivity to exogenous ABA (Bethke et al., 2006b). Taken together, these results suggest that NO can decrease ABA sensitivity. A possible effect of NO on ABA catabolism was consequently investigated. Seed treatment with NO donor enhanced CYP707A2 transcript and protein accumulation while the NO scavenger c-PTIO reduced CYP707A2 expression and reversed the NO donor effect (Liu et al., 2009). Thus, as for nitrate, NO was found to enhanced *CYP707A2* gene expression (Liu et al., 2009). These results consequently reinforce the assumption that nitrate does not affect seed dormancy on its own but rather act through NO biosynthesis.

A rapid accumulation of NO, possibly in the endosperm layer, during the first stage of *Arabidopsis* seed imbibition is required for rapid ABA catabolism and breaking of dormancy (Liu et al., 2009). A similar NO accumulation during imbibition was also observed in germinating seeds from other species (Simontacchi et al., 2007). Recently, NO was suggested to act upstream of GAs in a signaling pathway leading to vacuolation of protein storage vacuoles in aleurone cells, a process inhibited by ABA (Bethke et al., 2007a). However, the growth of isolated embryos was unaffected by NO donor or scavengers. Thus, the endosperm layer, proposed as the primary determinant of seed dormancy in *Arabidopsis*, was proved to perceive and respond to NO, and suggested as its main site of synthesis and action in seeds (Bethke et al., 2007a). Apart from its effect on the hormonal balance, it has been speculated that NO might accelerate the flux towards the pentose phosphate pathway (PPP) by indirectly increasing the oxidation of nicotinamide adenine dinucleotide phosphate (NADPH; Hendricks and Taylorson, 1974; Bethke et al., 2007b). Interestingly, the oxidation of NADPH by *S-*nitrosoglutathione (GSNO) in the presence of thioredoxin reductase and thioredoxin was demonstrated, releasing glutathione (GSH) and NO (Nikitovic and Holmgren, 1996). In addition, the involvement of the hemoglobin/NO in the oxidation of NADPH has been proposed (Igamberdiev and Hill, 2004). An increase in glucose catabolism via PPP could in turn promote dormancy release (Roberts and Smith, 1977).

As a conclusion, NO is a likely player of a signaling pathway that promotes loss of dormancy and has been suggested to behave as an endogenous regulator of this process. However, the direct targets of NO in seeds remain unclear. Nonetheless, some consequences of NO accumulation on seed metabolism have been highlighted and pinpoint an implication in the regulation of ABA metabolism.

#### Reactive oxygen species and no crosstalk in the control of seed dormancy and germination

In parallel to NO, ROS have emerged as key players in the control of seed dormancy and germination (Bailly, 2004; Bailly et al., 2008). In cells, ROS can be generated by specific enzymatic reactions or as by-products of the metabolism. Depending on their concentration, ROS may have positive signaling effects including the promotion of dormancy release and germination or detrimental consequences (Liu et al., 2010; Leymarie et al., 2012). Accordingly, it has been proposed that the amount of ROS generated upon seed imbibition should fall within a defined “oxidative window” for germination to occur (Bailly et al., 2008). Below this window, ROS levels would be too low to promote dormancy alleviation while above, oxidative damages would be predominant.

Recently, it has been proposed that ROS might coordinate the reduction of ABA-imposed dormancy with the onset of GA-stimulated germination (Liu et al., 2010). More precisely, exogenous hydrogen peroxide (H_2_O_2_) was shown to enhance ABA catabolism and GA biosynthesis during seed imbibition. As NO scavenger efficiently reversed H_2_O_2_-mediated induction of *CYP707A* genes but had no effect on the stimulation of GA biosynthesis, NO was proposed to act downstream of H_2_O_2 _ in enhancing ABA catabolism. *In vivo*, both H_2_O_2 _ and NO appeared to accumulate rapidly and concomitantly upon imbibition and to precede the induction of ABA catabolism/GA biosynthesis (Liu et al., 2010).

**FIGURE 1:**
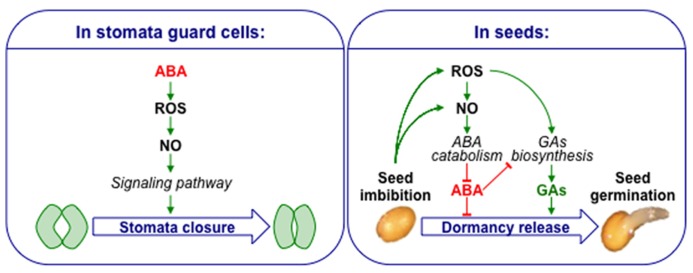
**Contrasting models showing ROS, NO, and ABA crosstalk in stomata guard cells and seeds.** ABA increases ROS and NO level in guard cells leading to ABA-dependent stomatal closure. Seed imbibition leads to ROS and NO accumulation. ROS up-regulate ABA catabolism through NO, and also GA biosynthesis. A high concentration of ABA also inhibits GA biosynthesis, but a balance of these two hormones jointly controls seed dormancy and germination.

In stomatal guard cells, one of the well-established signaling pathway for ABA-induced stomatal closure involve the successive accumulation of ROS and NO, acting as secondary messengers of ABA signal (Neill et al., 2008; Simontacchi et al., 2013). Even though similar actors are present in seeds, the picture is obviously quite different as both ROS and NO counteract ABA-inhibition of seed germination. This clear distinction highlights the specificity of seed physiology (**Figure 1**). The exact interplay between reactive nitrogen and oxygen species is always difficult to interpret due to the non-enzymatic reactions susceptible to occur and the molecular consequences they might have.

#### Nitric oxide and germination vigor

Nitric oxide is well known to play a dual role in stress responses in plants (Corpas et al., 2011). In particular, NO can directly scavenge certain ROS such as superoxide anions and lipid-derived radicals and was shown to stimulate antioxidant enzymes thereby limiting oxidative damages. However, uncontrolled NO accumulation referred to as nitrosative stress can have detrimental consequences.

In seeds, pharmacological experiments highlighted that NO did not significantly influence the germination of non-dormant (fully after-ripened) *Arabidopsis* seeds under optimal conditions (Bethke et al., 2006a). However, in rice seed, NO was proved to enhance germination by stimulating the transcription of the plasma membrane intrinsic protein (PIP) genes encoding water channels (Liu et al., 2007). In addition, several studies suggested that NO could participate in the tolerance to abiotic stresses during seed germination (Sirova et al., 2011). In particular, NO was demonstrated to delay programmed cell death of barley aleurone cells by promoting the activity of antioxidant enzymes (Beligni et al., 2002). In addition, SNP, commonly used as NO-donor, was shown to alleviate heavy metal stress during seed germination of wheat (Hu et al., 2007), lupin (Kopyra and Gwóźdź, 2003) and rice (Péres da Rocha Oliveiros Marciano et al., 2010). Seed pre-incubation (seed priming) with SNP was also proved to increase salt stress tolerance in wheat (Duan et al., 2007; Zheng et al., 2009). Finally, two recent papers on *Arabidopsis* reported an enhanced sensitivity of mutants with reduced NO accumulation (*atnoa* and *nia1nia2*) to salt and osmotic stress (Zhao et al., 2007; Lozano-Juste and Leon, 2010). In the few cases where it was examined, stress tolerance was associated to increased antioxidant activity. NO could therefore play a key role in germination vigor that could result from its crosstalk with ROS. NO and superoxide rapidly combine to form peroxynitrite (ONOO^-^), a selective oxidant able to reacts with most biological molecules. Peroxynitrite modifies protein tyrosine to create nitrotyrosines, leaving a footprint detectable *in vivo* (Vandelle and Delledonne, 2011). However, up to now, only indirect evidences support this assumption in *Arabidopsis* seeds and none investigated the underlying mechanisms associated to the increased tolerance observed. Overall, NO could play a pivotal role in the sensing of environmental conditions appropriate for seed germination.

## CONSEQUENCES OF NITRIC OXIDE CHEMISTRY ON THE INVESTIGATION ON ITS SIGNALING IN SEEDS

### SPECIFICITIES OF NITRIC OXIDE CHEMISTRY AND SIGNALING

Nitric oxide (NO^•^) is an uncharged, gaseous and lipophilic free radical that can readily diffuses across biological membranes. Thus, NO can interact with numerous distinct molecules in plant cells and therefore acts as a signaling element. Free NO^•^ is a transient compound displaying a high reactivity toward other free radicals (e.g., superoxide anion) and transition metal ions (e.g., iron; Wink and Mitchell, 1998). Thus, upon production, released NO can adjust to the cellular redox environment leading to the formation of diverse biologically active compounds collectively referred to as reactive nitrogen species (RNS). RNS include nitrosonium (NO^+^) and the nitroxyl anion (NO^-^), respectively resulting from a gain or loss of one electron by NO and peroxynitrite (ONOO^-^) product of the reaction of NO with superoxide anion radical ($o_{2}^{-}$ ; Stamler et al., 1992b). Oxidation reactions in the presence of molecular oxygen (O_2_) can also lead to nitrogen dioxide (NO_2_), nitrous anhydride (N_2_O_3_), ${NO}_{2}^{-}$ and ${NO}_{3}^{-}$ generation. All these molecules differ in reactivity toward the range of NO biological targets. Their differential production can thus orient and/or alter the message mediated by NO. Under physiologic conditions, a strict control of NO content is required to maintain proper cellular functions. High accuracy in signaling events can only be achieved through a tight spatio-temporal control of the intracellular levels of the messengers. Therefore, the balance between NO production and elimination (conversion or storage) is of major importance in determining the biological effects of this radical (Besson-Bard et al., 2008; Moreau et al., 2010; Baudouin, 2011). As for ROS, the chemical reactivity of NO (and associated RNS), make it a particular signal element which can readily interact with a wide range of targets (e.g., proteins, lipids) rather than interact with “dedicated” receptors (Kalyanaraman, 2004; Besson-Bard et al., 2008). The signal mediated by NO can belongs to transduction pathways or be associated with nitrosative stress depending on the biological environment.

### NITRIC OXIDE SYNTHESIS AND HOMEOSTASIS IN PLANT SEEDS

Distinct pathways have been proposed to account for NO generation in plant cells (Reviewed in Gupta et al., 2011a). However, the reactions and enzymes involved are still a matter of debate and the relative contribution of these NO biosynthesis pathways remains unclear in seeds (Reviewed in Arc et al., 2013). For instance, NR-catalyzed reduction of nitrite into NO in the cytosol is presumably the most documented reaction but its relevance in seeds is controversial. Instead, nitrite reduction was suggested to occur either via non-enzymatic reactions especially within the apoplasm possibly next to the endosperm layer (Bethke et al., 2004a) or in hypoxic mitochondria (Igamberdiev et al., 2010; Gupta and Igamberdiev, 2011). Alternatively, NO synthesis could result from oxidative reactions from hydroxylamine, polyamines or L-arginine (L-Arg) pathways. NO can also be “stored” through its interaction with diverse molecules. Indeed, NO can react with reduced GSH or thiol groups leading to the reversible formation of *S*-nitrosothiols (e.g., GSNO, *S*-nitrosylated proteins). GSNO was suggested to constitute a storage and transport form for NO, even in seeds (Sakamoto et al., 2002; Catusse et al., 2008).

### NITRIC OXIDE DETOXIFICATION BY NON-SYMBIOTIC HEMOGLOBINS

Hemoglobins are well known in the animal kingdom for their role as oxygen carrier. In plants, non-symbiotic hemoglobins (nsHb) are divided into two main classes with distinct properties. Class 2 nsHb are the only proteins with an affinity for oxygen fitting with a direct role in oxygen storage and supply (Spyrakis et al., 2011; Vigeolas et al., 2011). Contrarily, the very high affinity for oxygen (in the order of 1–2 nM) displayed by class 1 nsHb is not compatible with such function (Dordas, 2009; Gupta et al., 2011b; Hill, 2012). The plant nsHb1 can act as NADPH-dependent dioxygenase metabolizing NO into nitrate (Igamberdiev and Hill, 2004; Perazzolli et al., 2004). Under hypoxia, NO can be generated from nitrite by deoxyhemeproteins within the mitochondria. Then, nsHb1 and NR can allow the NADPH-dependant re-oxidation of NO into nitrite in the cytosol. As NO can reversibly inhibits cytochrome c oxidase, the reaction between NO and nsHb1 is part of a dynamic equilibrium allowing a tight adjustment of the cellular energy and redox state to oxygen availability (Hebelstrup et al., 2007). These reactions constitute the so-called hemoglobin-NO cycle (Igamberdiev et al., 2010). Furthermore, nsHb1 protein also participates in NO scavenging and therefore NO homeostasis. Accordingly, modulation of nsHb1 expression in plants was shown to directly impact NO levels at distinct developmental stages including seeds (Hebelstrup and Jensen, 2008; Thiel et al., 2011) and in diverse environmental conditions (Dordas, 2009; Cantrel et al., 2011). Thus, despite putative other functions, like CO binding (Hill, 2012), the use of transgenic lines with altered AHb1 expression proved to be a valuable tool to highlight NO implication in physiological processes and stress tolerance.

The over-expression of *Arabidopsis* nsHb1, AHb1 (also named GLB1 or AtHb1 in other studies; At2g16060) in seeds resulted in a pre-adaptation to stress with the repression of energy consuming pathways, modulation of hormone metabolisms (ABA, SA, auxin, ethylene⋯) and reduced NO emission under transient hypoxia (Thiel et al., 2011). Overall, this leaded to a more efficient allocation of energy resources in seeds resulting in higher weight of mature transgenic seeds (Thiel et al., 2011). Thus, this study highlighted an impact of AHb1 over-expression on the nitrosative stress induced by hypoxia and possibly on NO mediated signaling during seed maturation. However, in dry mature wild-type (WT) seeds, neither nsHb1 protein nor the corresponding mRNA has been detected so far, instead both accumulated during seed imbibition suggesting a crosstalk between nsHbs1 and NO in the germination process (Duff et al., 1998; Ross et al., 2001; Hebelstrup et al., 2007; Matilla and Rodriguez-Gacio Mdel, 2013). Indeed, the NO dioxygenase activity of nsHb1 may also have a significant impact on seed physiology. Importantly, NO accumulation upon water uptake seems to precede nsHb1 induction (Hebelstrup et al., 2007; Liu et al., 2009; *Arabidopsis* EFP-browser dataset, Winter et al., 2007). The shift between the induction of NO release and nsHb1 accumulation could delimit a short time window during which NO-mediates its effect on ABA catabolism thereby allowing dormancy release before the re-establishment of NO homeostasis by nsHb1 as required to avoid nitrosative stress.

Previous studies relying on modulation of nsHb1 expression in seeds mainly focused on seed maturation (Thiel et al., 2011; Vigeolas et al., 2011). Yet, to date, the link between the NO-related AHb1 function and physiology of seed germination (dormancy, germination vigor, longevity) has never been addressed.

### DETECTION AND STUDY OF NITRIC OXIDE IN SEEDS

The investigations on the mode of action of NO in plant cells still suffer from several technical limitations. Indeed, the improvement of NO detection and quantification, pharmacological approaches and biochemical assay for the analysis of NO-induced PTMs are still required.

#### Pharmacological experiments

Most of the known implications of NO in plant physiology were first highlighted through pharmacological experiments employing NO donors and/or NO scavengers (Bethke et al., 2011). Indeed, due to the toxicity, reactivity, and gaseous state of NO, direct application is not easy to carry out in the laboratory. Thus, a plethora of compounds known to generate NO are preferentially used instead. All these molecules differ by their characteristics of NO release (kinetic, amount, light-dependency) and can thus lead to contrasted results (Planchet and Kaiser, 2006b). Used in aqueous solutions, NO donors can lead to nitrogen oxides production. In addition, certain of these chemicals are complex molecules with potential side products. For instance, the photolysis of SNP was proved to release more cyanide than NO. Indeed, cyanide may actually be the active compound when applying SNP to seeds (Bethke et al., 2006a). Conversely, the widely used derivatives of PTIO such as c-PTIO are thought to be relatively specific NO scavengers (Akaike et al., 1993): PTIO + NO → PTI + NO_2_. However, the reaction products including PTI may have undesirable side effects in cells (Planchet and Kaiser, 2006a). In a general way, when using NO donors or scavengers, the potential effect of all generated compounds should always be taken into account. The demonstration of opposite effects of NO donors and NO scavengers in a given physiological process is usually considered as a reliable evidence of NO implication.

#### Methods available for the detection and quantification of nitric oxide in seeds

In animal cells, the absence of nitrate reduction pathways allows the use of assay based on nitrogen oxides, especially nitrite, quantification to evaluate NO production (nitrate and nitrite being considered as by-products of NO production and subsequent oxidation). In plant, such methodology is excluded due to the existence of an active nitrate assimilation pathway responsible for most of nitrite production. Consequently, distinct other methodologies have been applied including fluorescent probes based detection, EPR spectroscopy, electrochemistry, ozone based chemiluminescence, laser photoacoustic, mass spectrometry and the oxyhemoglobin assay. A short discussion on some of these techniques is provided below, for a complete review refer to (Vandelle and Delledonne, 2008; Bethke et al., 2011; Mur et al., 2011).

Several distinct fluorescent probes can be used to investigate NO biosynthesis or release by a given tissue. The diaminofluoresceins (DAF; DAF-FM, 4-amino-5-methylamino-2′,7′-difluorofluorescein) or the diaminorhodamine 4M (DAR-4M) and their cell permeable forms DAF diacetate (DAF-2DA, DAF-FM DA) and DAR-4M acetoxymethyl ester (DAR-4M AM) are the most commonly used (Kojima et al., 1998, 2001). These probes are sensitive (up to the nM range) but suffer from a serious lack of specificity. Indeed, they do not directly react with NO but with its main oxidation product N_2_O_3_. Thus, the fluorescence intensity could also depend on the rate of NO oxidation. As the non-enzymatic oxidation of NO requires oxygen, these fluorescent probes cannot be used under anoxia. Finally, numerous distinct compounds were reported to affect DAF-T fluorescence *in vivo* including ascorbate and dehydroascorbate (Vandelle and Delledonne, 2008). Nonetheless, N_2_O_3_ detection with DAF-FM was successfully applied on *Arabidopsis* seeds but required to remove the seed testa (Liu et al., 2009).

Electron paramagnetic resonance spectroscopy is a more specific method that can be applied to the direct detection of radical species including NO both *in vitro* and *in vivo*. However, in order to increase its sensitivity, EPR spectroscopy is often associated with the use of spin-traps, molecules that can react with NO and enhance its EPR signal. This technique has been successfully applied to the detection and quantification of NO in embryonic axes homogenates from soybean and sorghum (Caro and Puntarulo, 1999; Simontacchi et al., 2004). However, NO detection from intact seed tissues, eventually supplemented with a spin trap remains a technical challenge as it would require a sufficiently high production to reach the sensitivity threshold.

Another widely used approach is based on the chemiluminescent reaction between gaseous NO and ozone. This technique can allow the direct quantification of NO release from a tissue placed in a sealed compartment under a gaseous flux driving the gas released in the environment to an analyser. NO-specific electrodes are also available but are also difficult to apply to the study of the tiny *Arabidopsis* seeds. They could only be useful to assess the amount of NO released by the seeds in their environment.

Overall, despites all the existing techniques, an accurate detection and quantification of NO generation in plant tissue remain difficult. In addition, most techniques require preparation steps or experimental conditions that can lead to undesirable signal. Thus, as for the pharmacological experiments, a cross validation with at least two distinct quantification methods is highly recommended (Gupta and Igamberdiev, 2013). In case of *Arabidopsis* seeds, the size and characteristics of the mature seeds represents significant technical constraints to an accurate and specific detection/quantification of NO levels by the methodologies currently available.

#### Genetic resources for the study of nitric oxide production and signaling

The genetic resources available to investigate NO signaling remain restricted due to our limited actual knowledge of NO biosynthesis pathways in plants. Thus, most of the studies rely either on a pharmacological approach (as discussed previously) or on mutants affected in NO availability although their NO levels are not always explained. Some mutants somehow related to NO homeostasis in plants (e.g., *nia1nia2, gsnor, atnoa1)* have been associated to seed phenotypes. However, the interpretation of these phenotypes is often difficult and requires a lot of caution.

Nitrate reductase, being the only identified enzyme proven to be directly involved in NO biosynthesis, NR-deficient mutants has been extensively used, especially the G′4–3 mutant in *Arabidopsis* (Wilkinson and Crawford, 1993). However, NR-deficiency causes important perturbation of nitrogen metabolism and a significant nitrate accumulation resulting in a pleiotropic phenotype (Alboresi et al., 2005). Consequently, it is difficult to establish a direct link between nitrate-related phenotypes and reduced NO production by NR-NiR activity. Moreover, the high nitrate levels could lead to an enhanced NO-independent nitrate-mediated signaling (Alboresi et al., 2005). Contradictory results have been published regarding G′4–3 seeds physiology (Alboresi et al., 2005; Lozano-Juste and Leon, 2010).

Several other mutants known as affected in NO levels have also been used to investigate NO signaling in *Arabidopsis*. Mutants associated to reduced NO levels include NO-Associated 1 (*atnoa1*, At3g47450; Guo et al., 2003) and prohibitin 3 (*phb3*; At5g40770; Wang et al., 2010) while one mutant with enhanced endogenous NO levels was identified as the phosphoenolpyruvate/phosphate translocator chlorophyll a/b binding protein underexpressed 1/NO overproducer 1 (*cue1/nox1*; At5g33320; He et al., 2004). The exact relation between the function of the corresponding proteins and the NO levels in these mutants has not been clearly elucidated yet. Most of these mutants have strong phenotypes but, *phb3* and *cue1/nox1* have not been investigated for seed phenotypes. However, the *atnoa1* mutant has been more studied as it was first proposed as encoding a NO synthase (NOS)-like protein based on sequence similarity with an hypothetical snail NOS and subsequent characterization of a corresponding mutant displaying reduced NOS activity in leaves and lower NO levels in roots (Guo et al., 2003). However, further experiments excluded a direct role for this protein in NO synthesis. Instead, it was later identified as a GTPase. The *atnoa1* mutant seeds were associated with a slightly increased dormancy and a hypersensitivity to salt and osmotic stresses (Zhao et al., 2007; Lozano-Juste and Leon, 2010).

Alternative strategies have been developed to get around the known limitations and pursue the investigations on NO signaling in plants. Promising examples include the use of transgenic lines with altered hemoglobin expression (Perazzolli et al., 2004) and the over-expression of rat neuronal NOS in *Arabidopsis* (Shi et al., 2012). Both strategies already led to significant results even thought all putative side consequences, apart from NO levels alteration, must be considered with extreme caution.

## MOLECULAR TARGETS OF NITRIC OXIDE IN SEEDS

**FIGURE 2:**
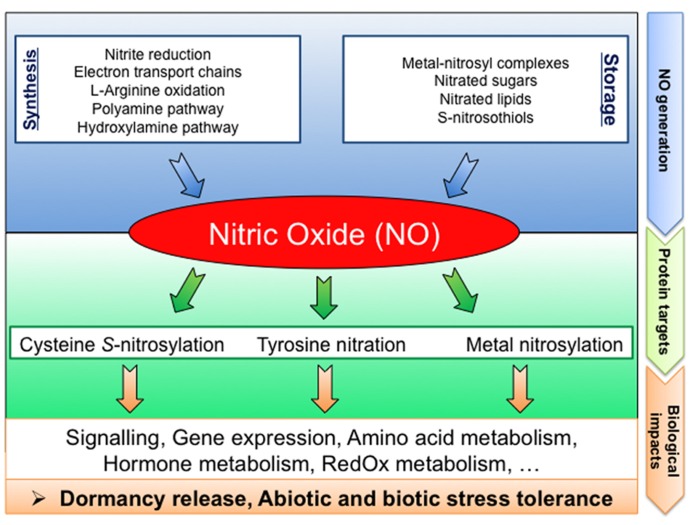
**Schematic diagram summarizing NO signaling in seeds: from generation to targets.** NO can be produced by various biosynthesis pathways or released from NO-storage compounds. Proteins are preferentially targeted by NO resulting in PTMs (cysteine *S-*nitrosylation, tyrosine nitration, and metal nitrosylation). These NO mediated PTMs modulate the protein functions, leading to strong impacts on cell metabolism thereby affecting seed physiology.

Aside from the long lasting question concerning the relevant NO sources in seeds, the re-constitution of NO signaling pathways require the identification of the NO biological targets. Yet, direct molecular targets of NO remain poorly documented in plants. NO could regulate physiological processes by affecting gene transcription. Indeed, several NO-regulated genes, involved in different functional and biological processes, have previously been described (Huang et al., 2002; Polverari et al., 2003; Parani et al., 2004; Grun et al., 2006; Palmieri et al., 2008; Besson-Bard et al., 2009). Furthermore, NO can bind to transition metals of metalloproteins (metal nitrosylation) or cause protein PTMs such as cysteine *S-*nitrosylation or tyrosine nitration (**Figure 2**; Moreau et al., 2010; Arc et al., 2011).

### PROTEIN *S-*NITROSYLATION IN SEEDS

Nitric oxide-mediated *S-*nitrosylation of cysteine thiol groups within polypeptide chains is a likely mechanism by which NO may function in signaling processes (Stamler et al., 1992a; Jaffrey et al., 2001). *S-*nitrosylation consists in the covalent attachment of a NO moiety to a reactive cysteine thiol resulting in the formation of a *S-*nitrosothiol group (*S-*NO). In animal systems, regulation of specific proteins by *S-*nitrosylation is an intensively investigated PTM. This PTM, which is thought to be particularly labile, is associated with a precise spatio-temporal regulation and can potentially result in the activation or inactivation of targeted proteins (Hess et al., 2005). It occurs mainly through non-enzymatic reactions being dependent on the physiochemical environment of the protein cysteinyl residues and the proximity of susceptible proteins to NO production sites in cells (Lindermayr and Durner, 2009). On the contrary, protein de-nitrosylation seems to be catalyzed by several enzymes, such as thioredoxins (Trxs) or Cu/Zn superoxide dismutases, as well as by reducing metals and intracellular reducing agents (Lindermayr and Durner, 2009). In fact, because of its selectivity toward protein targets, *S-*nitrosylation may represent a general pathway for modulating protein structure/function, analogs to protein phosphorylation (Spickett et al., 2006). Up to now, only few intracellular *S-*nitrosylated proteins have been identified in plants (Lindermayr et al., 2005; Tanou et al., 2009; Astier et al., 2011; Lounifi et al., 2012). A recent and promising example is the NO-mediated modulation of auxin signaling through the *S-*nitrosylation of the TIR1 auxin receptor. This PTM of TIR1 promotes its interaction with Aux/IAA repressors thereby facilitating their degradation (Terrile et al., 2012). Moreover, NO and ethylene act antagonistically in fruit ripening through inhibition of enzymes involved in ethylene production by *S-*nitrosylation (Manjunatha et al., 2012). In contrast, NO and ethylene act synergistically in seed dormancy release but the underlying molecular mechanisms are still unknown (Gniazdowska et al., 2010b; Arc et al., 2013). Due to the limited permeability of most of their outer layers, seeds can experience hypoxia (Borisjuk and Rolletschek, 2009). Consequently, a fine regulation of oxygen consumption is necessary. This seems to be achieved through NO-mediated inhibition of seed mitochondrial activity (Borisjuk et al., 2007). Consequently, NO-related protein modifications are likely to be increased in seed mitochondria and therefore to play an important role in regulating the activity of these organelles. Many *S-*nitrosylated proteins identified in plants are implicated in metabolic processes (Lindermayr et al., 2005; Abat et al., 2008; Romero-Puertas et al., 2008; Abat and Deswal, 2009; Tanou et al., 2009; Palmieri et al., 2010) suggesting that NO could participate in the regulation of the energy status of the seeds. In agreement, a β-subunit of the mitochondrial ATP synthase complex was found to be *S-*nitrosylated in dry *Arabidopsis* seeds (Arc et al., 2011). Since a homologous protein was shown to be inactivated by *S-*nitrosylation in alcoholic fatty liver of rats (Moon et al., 2006) and more recently in pea leaves mitochondria (Camejo et al., 2013), the seed mitochondrial ATP synthase activity might be inhibited by this NO-mediated PTM. Further experiments are required to assess this hypothesis.

In wheat seeds, a parallel increase in NO and protein *S-*nitrosylation was reported during *sensu stricto* germination (Sen, 2010). Noteworthy, seed treatments with NO promoted desiccation tolerance, in the recalcitrant species *Antiaris toxicaria*, by limiting protein carbonylation and enhancing protein *S-*nitrosylation (Bai et al., 2011).

### PROTEIN NITRATION IN SEEDS

Tyrosine nitration consists in the addition of a nitro group (–NO_2_) resulting in an alteration of diverse protein functions. The very fast reaction between NO and O_2-_ gives rise to peroxynitrite (ONOO^-^) which is considered as a potent oxidizing and nitrating agent (Ducrocq et al., 1999; Abello et al., 2009). Tyrosine nitration is consequently predominantly observed in states prone to the concomitant release of NO and ROS. Until recently, tyrosine nitration was considered as being irreversible suggesting that the presence of nitrotyrosine in proteins represents a footprint of nitrosative stress. However, increasing evidence suggests the existence of a de-nitration mechanism *in vivo* (Abello et al., 2009). Protein nitration can result in an alteration of diverse protein functions (Alvarez et al., 2011; Melo et al., 2011; Jacques et al., 2012) and could enhance protein sensibility to proteolytic degradation via the proteasome (Abello et al., 2009). Thus, protein nitration would be more than a biological marker of nitrosative stress and could participate in protein turnover or signal transduction in plants (Corpas et al., 2008, 2009; Ischiropoulos, 2009). A single study has been carried out on seeds, more precisely on sorghum embryonic axes (Jasid et al., 2008). This work revealed the appearance of several nitrated proteins upon seed imbibition. A recent study based on immunoprecipitation with an anti-3-nitrotyrosine antibody and subsequent analysis by shotgun liquid chromatography–mass spectrometry (LC-MS/MS) led to the identification of 127 proteins putatively targeted by this PTM in protein extracts from *Arabidopsis* seedlings (Lozano-Juste et al., 2011). Among this important list, a few candidates were further confirmed by additional experiments. Among these numerous putative targets of tyrosine nitration were a few proteins with known implications in seed physiology. For instance, the molybdenum cofactor (MoCo) sulfurase ABA3 (At1g16540) was among these candidates. ABA3 is involved in the last step of ABA synthesis (Mendel, 2007). Thus, the inactivation of ABA synthesis by this PTM might contribute to the control of dormancy release and germination vigor. Overall, nitration may be more than a biological marker of nitrosative stress and could participate in protein turnover or signal transduction in plants (Corpas et al., 2009; Ischiropoulos, 2009). In seeds, the concomitant generation of NO and ROS upon imbibition could lead to enhanced peroxynitrite formation thereby improving tyrosine nitration. Therefore, protein tyrosine nitrations appear likely to occur in this context and in lights of the discussed examples could be of paramount importance.

## CONCLUSIONS AND PROSPECTS

Most of the analysis published up to date pinpoint ABA content as a major determinant of dormancy release or maintenance. It appears that the decision to pursue the transition toward germination or maintain a dormant state can be taken during seed imbibition depending on environmental parameters. Thus, the control of ABA levels and sensitivity during early imbibition appears of paramount importance. During this phase, both NO and ROS accumulation has been reported. The intensity of the generation of these radicals could depend on both endogenous and environment cues. In turn, the interplay between ROS and RNS would determine both the extent of ABA catabolism (via the regulation of *CYP707A2 *expression for instance) and the sensitivity to this hormone. As a result, theses reactive species could determine the kinetics of ABA degradation and the threshold below which ABA content should fall for germination to occur. As the *de novo* protein synthesis is low during the first hours upon imbibition these effects could be mainly modulated *via* non-enzymatic protein PTMs such as carbonylation, nitration and/or *S-*nitrosylation. Still, both ROS and RNS accumulation can also lead to detrimental damages. Thus, we believe that the concept of “oxidative window” for seed germination should be extended to include NO and associated RNS.

However, despite a general consensus regarding NO importance in seed physiology, the pathways involved in its biosynthesis remain uncertain. This observation presumably reflects the complexity of the regulation of NO biosynthesis in plants. Indeed, multiple different endogenous sources all potentially depending on environmental and/or molecular parameters may contribute to NO accumulation in seeds. Moreover, the relevant reactions in seeds may be significantly different from those described at other physiological stages including the non-enzymatic reactions that may occur in the apoplast next to the aleurone layer (Bethke et al., 2004a). To discriminate between the relative contribution of the distinct known NO sources, accurate determination of NO content in seeds and especially during imbibition appears absolutely required. However, the relatively low amount of NO released under physiological conditions and the drawbacks of the techniques currently available makes NO measurement a very challenging issue. In any case, an unambiguous confirmation of NO accumulation in the seed endosperm and/or embryo appears as a priority to consolidate the available evidences and determine the seed NO content.

In a similar way, we are firmly convinced that NO-related PTMs, namely tyrosine nitration and cysteine *S-*nitrosylation, can explain the effect of NO in seeds though this assumption is not totally confirmed yet. Indeed, the detection and identification methods for both cysteine *S-*nitrosylation and tyrosine nitration proved difficult to apply on seeds most presumably due to the low abundance of modified proteins and/or the limited stability of the modifications. Nonetheless, these two PTMs represent very seducing models to explain the roles ascribed to NO in seeds. The characterization of NO-targeted proteins in various seed physiology context will undoubtedly reveal new area of research to explore for understanding the control of germination.

## Conflict of Interest Statement

The authors declare that the research was conducted in the absence of any commercial or financial relationships that could be construed as a potential conflict of interest.
